# Gender Differences in Laser Acupuncture—Results of a Crossover Study with Green and Yellow Laser at the Ear Point Shenmen

**DOI:** 10.3390/medicines5010024

**Published:** 2018-03-15

**Authors:** Daniela Litscher, Junying Wang, Gerhard Litscher, Guangzong Li, Peggy Bosch, Maurits van den Noort, Lu Wang

**Affiliations:** 1 TCM Research Center Graz, Research Unit of Biomedical Engineering in Anesthesia and Intensive Care Medicine, and Research Unit for Complementary and Integrative Laser Medicine, Medical University of Graz, 8036 Graz, Austria; daniela.litscher@medunigraz.at (D.L.); snshine@163.com (G.L.); lu.wang@medunigraz.at (L.W.); 2 Institute of Acupuncture-Moxibustion, China Academy of Chinese Medical Sciences, 100700 Beijing, China; wjyanguning@aliyun.com; 3 Donders Institute for Brain, Cognition and Behaviour, Radboud University, 6525 Nijmegen, The Netherlands; p.bosch@donders.ru.nl; 4 Research Group of Pain and Neuroscience, Kyung Hee University, 02447 Seoul, Korea; info@mauritsvandennoort.com

**Keywords:** acupuncture, green laser, yellow laser, ear acupuncture, Shenmen, gender, blood pressure, heart rate, heart rate variability, temperature

## Abstract

**Background:** One of the most commonly used auricular acupuncture points selected for different pain treatment regimens is Shenmen. This point on the ear has been recognized as having a wide number of applications, as found by scientific investigation. **Methods:** Within this crossover study, the ear acupoint Shenmen was stimulated with two different kinds of laser (green, 532 nm and yellow, 589 nm) in 22 healthy volunteers (13 female, 9 male; mean age ± SD = 25.3 ± 4.1 years; range 21–36 years). Both green and yellow lasers were used for 15 min in the same volunteers in two different sessions. **Results:** The most prominent finding was that systolic blood pressure decreased significantly (*p* = 0.048) after yellow laser stimulation. Heart rate also decreased significantly (*p* < 0.001), whereas heart rate variability ratio low frequency (LF)/high frequency (HF) (*p* < 0.001) increased. The effects were significantly more pronounced in females than in males. In addition, the temperature was measured, and temperature increases were demonstrated at different locations on the ear using imaging methods. **Conclusions:** This study shows evidence of the effect of auricular laser acupuncture. However, a comparison with other publications was impossible because this is the first study using green and yellow laser stimulation on the ear.

## 1. Introduction

Laser acupuncture is a scientifically well-investigated method for the non-invasive stimulation of acupuncture points and has proven to be clinically effective in both patients with needle phobia and children [1,2]. Laser acupuncture is an excellent way to provide effective treatment without needles. However, there is currently a lack of extensive fundamental studies in order to be able to describe the effects of different wavelengths and the influence of different dose effects on physiological and pathophysiological parameters in humans.

The question “which wavelength should be used in laser acupuncture” is sometimes related to the question “how deep does light penetrate human tissue”. It is well known that red laser light has a deeper penetration depth than violet, blue, green, or yellow. Infrared light is not visible, but some authors have demonstrated that it penetrates human tissue at least as deep as visible red light. Within this study, we have used green (532 nm) and yellow (589 nm) laser light because for ear acupuncture we do not need a very high penetration depth [1].

The original aim of the present randomized crossover study was to analyze for the first time the possible effects of a single-point laser ear acupuncture (Shenmen, right ear) with green and yellow lasers on physiological parameters of subjects. However, the gender-specific evaluation also brought very interesting results, although the original study design was not designed primarily for their analysis.

## 2. Materials and Methods 

### 2.1. Laser Acupuncture

**Figure 1:**
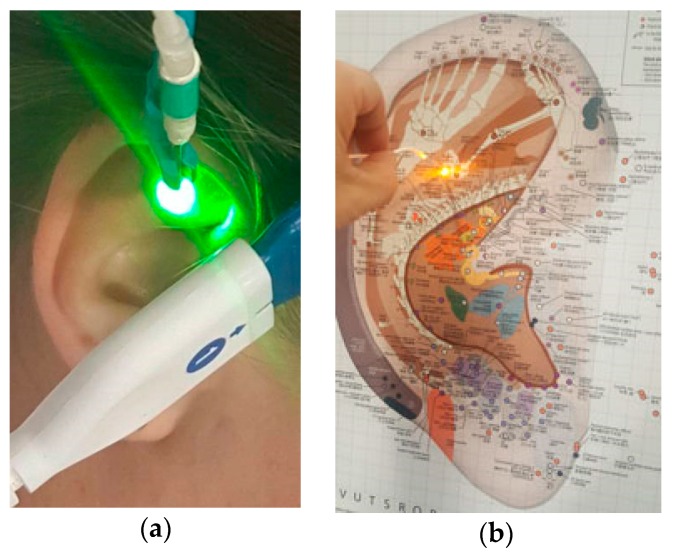
Green (**a**) and yellow (**b**) ear acupuncture laser at the ear point Shenmen.

The radiation of a green (wavelength: 532 nm, output power: 5 mW, fiber core diameter: 500 μm) and a yellow (589 nm, 30 mW, 500 μm) laser (Weber Medical GmbH, Lauenförde, Germany) was coupled into an optical fiber and the so-called laser needle was attached to the distal end of the optical fiber (Figure 1). The dosage (energy density) for the green laser treatment was 2.3 kJ/cm^2^ and for the yellow laser it was calculated to be 13.8 kJ/cm^2^.

Acupuncture with a green laser (e.g., 532 nm) has so far not found a broad field of application. There are a few indications to use the method as a supplement to red, near-infrared, and violet laser stimulation within the framework of acupuncture. The penetration depth of the green laser (532 nm) is 0.5–1 cm [1].

The yellow laser is another option for laser acupuncture. Our research group is the first ever to study yellow light laser [1]. The production of yellow laser light is technically not easy. Usually, a laser consists of an infrared laser diode and a so-called combo crystal. This pair of crystals produces the visible laser light. In this process, the combo crystal receives the necessary energy from the infrared diode. For a green laser, one crystal produces laser light of 1064 nm and the other one doubles the frequency, which means that the wavelength is divided in half, resulting in 532 nm, i.e., green light. For yellow laser light to be produced, laser light of 1340 nm is necessary. If the frequency of this light was simply doubled, the emitted laser light would be red, but if red and green light is mixed, the result is yellow light. It is, however, a disadvantage that at 1340 nm only very little light is emitted, so the infrared diode needs to have a large power. Moreover, special filters have to be used in order to obtain the correct ratio of light at 1064 and 1340 nm. This makes the production expensive and complex, which is why yellow lasers are very expensive and, accordingly, rare [1]. The Shenmen point on the right ear was stimulated (see Figure 1). The point is located in the lateral third of the triangular fossa, at the crotch between the upper and lower antihelix. The acupuncture point is mainly used for insomnia, pain, and itching of the skin. Its effect is to soothe the mind, relieve pain, reduce stress, and reduce inflammation [3].

### 2.2. Subjects

A total of 22 volunteers with a mean age ± SD of 25.3 ± 4.1 years (13 female, 9 male, age range 21 to 36 years) were stimulated with both green and yellow lasers in two sessions each. The average stature of the subjects was 172.6 ± 7.9 cm, and the average body weight was 66.9 ± 14.0 kg. No person was under the influence of drugs. The non-invasive stimulation and registration of vital signals were approved by the local ethics committee and performed in accordance with the recommendations of the Helsinki Declaration of the World Medical Association. All subjects gave their written consent.

### 2.3. Procedure—Monitoring

**Figure 2:**
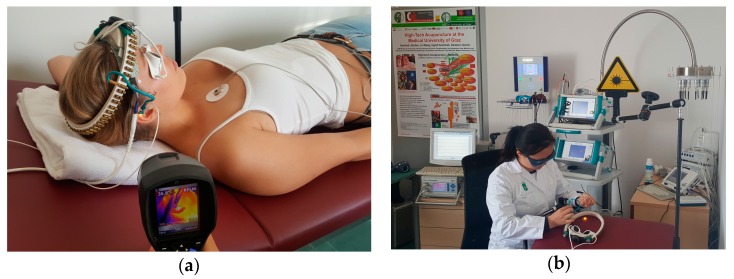
Yellow laser acupuncture at the ear point Shenmen (**a**) at the TCM Research Center of the Medical University of Graz (**b**).

**Figure 3:**
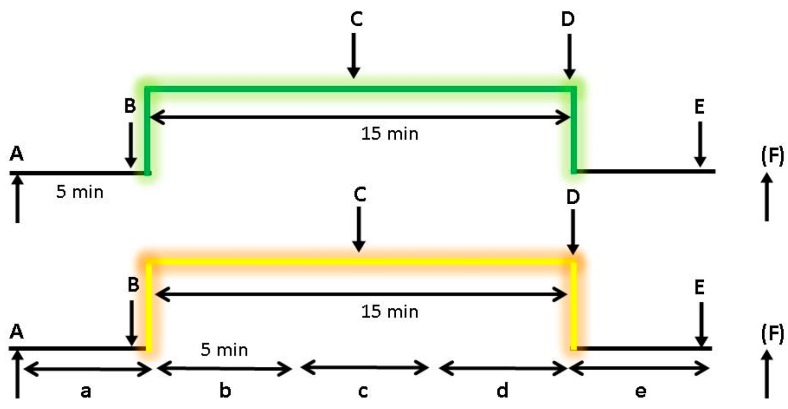
Measurement profile with analysis time intervals (a–e) and time points (A–F) for green and yellow laser stimulation. For the temperature measurement, an additional point (F; 15 min after point E) was used.

The measurement procedure was divided into two sections: One 15 min green and one 15 min yellow laser acupuncture were performed on the subjects in randomized order. A computerized random number generator was used to produce a randomization schedule employing simple randomization by an independent researcher, who was not involved in the recruitment, intervention, assessment, or statistical analysis. Ten subjects were assigned to the green laser first (7 female, 3 male) and 12 to the yellow laser first (6 female, 6 male). There was a break of at least 30 min between the two stimulation modalities. The study was conducted in the laboratory of the TCM Research Center of the Medical University of Graz, and the participants lay relaxed on a patient couch during the entire examination (see Figure 2). The procedure and the 5 min analysis sections are shown in Figure 3.

The monitoring included the following signals or parameters: Neurovegetative parameters (heart rate HR and heart rate variability HRV) were recorded with an HRV Medilog^®^ AR12 system (Huntleigh Healthcare, Cardiff, UK). This system is suitable for a monitoring period over 24 h. The sampling rate was 4096 Hz. Three Skintact Premier F-55 ECG electrodes (Leonhard Lang GmbH, Innsbruck, Austria) were attached to the thorax (see Figure 2a). The raw data were stored on special memory cards. HRV was measured as the percentage change in consecutive ventricular complexes in the ECG (RR intervals). The length of the RR intervals was analyzed within a certain time interval (5 min intervals a–e) (see Figure 3), and the HRV was determined by spectral analysis. The mean HR, the total power of HRV (HRV total), and the ratio of LF (low frequency) to HF (high frequency) were used as evaluation parameters, which are also parameters recommended by the Task Force of the European Society of Cardiology and the North American Society of Pacing and Electrophysiology [4]. The analysis was made in each of the five sections a–e (see Figure 3). The calculation of ECG power spectra is thought to provide information about the effects of the sympathetic and parasympathetic systems on HRV [1,4]. Early work demonstrated that several frequency bands in the HRV spectrum could be interpreted as physiologically relevant markers. Associated mechanisms include thermoregulation, which can be found in the very low frequency band, blood pressure, and respiratory effects [1,4].

The blood pressure (systolic BP sys and diastolic BP dia) and the mean blood flow velocity v_m_ in the right middle cerebral artery were respectively registered after an initial resting period of 10 min immediately before the start of the measurements and after completion of the measurement scheme (blood pressure: Mio-Star^®^, Migros, Vienna, Austria; Transcranial Doppler sonography: Smart Dop, DWL Elektronische Systeme, Sipplingen, Germany).

In addition, thermographic images of the ear (infrared camera Flir i5: Flir Systems Inc., Portland, USA) were taken at each of the measurement times A–F, and analyses of peripheral temperature-specific measurements at times A–E were performed (temperature monitor DRT4: Moor Instruments, Millwey, Axminster, England).

### 2.4. Statistical Analysis

The statistical analysis was carried out with the computer program SigmaPlot 13.0 (Systat Software, Chicago, IL, USA). An analysis of variance (one-way repeated measure ANOVA) was used. In addition, post-hoc analyses (Tukey test, Dunnett’s method) were carried out. The level of significance was set at *p* < 0.05.

## 3. Results

**Figure 4:**
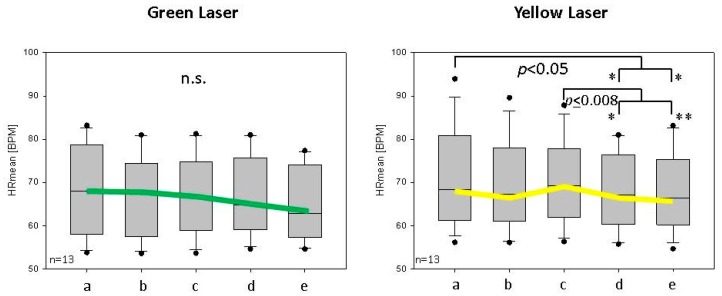
Box-plot of change in mean heart rate (HRmean in BPM (beats per minute)) values of all 13 female subjects before and after green and yellow laser acupuncture (phases a–e, see also Figure 3). The line in the box indicates the position of the median, the ends of the boxes define the 25th and 75th percentiles. The error bars show the 10th and 90th percentiles, and the dots represent “outliers”. Note the significance of the decrease in mean HR during (d) and after (e) yellow laser stimulation (* significant changes).

The HR values of all 22 subjects showed a significant decrease (*p* < 0.003) during and after yellow laser stimulation in sections d and e compared to baseline values a (see Figure 3). Significant differences were found for the 13 female subjects whose graphic presentation is shown in Figure 4. For green laser stimulation, the differences measured were not significant in both women and men, although the trend in comparison to yellow laser showed a similar behavior (decrease in HR during and after laser stimulation at the point Shenmen).

**Figure 5:**
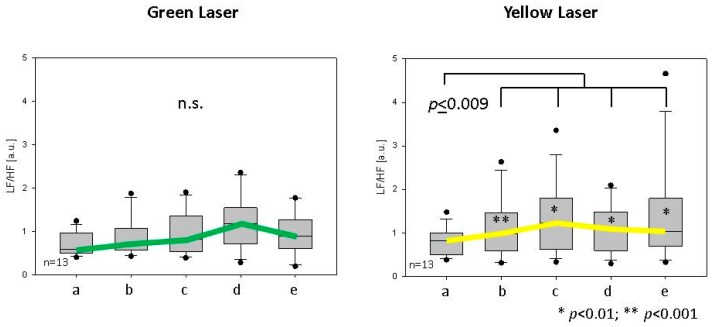
Heart rate variability: low frequency (LF)/high frequency (HF) ratio in women after green and yellow laser stimulation. Note the significant increase during (b–d) and after (e) Shenmen (ear point) irradiation with yellow laser light. For further explanations see also Figure 3 and Figure 4.

In terms of total power of heart rate variability (HRV total), there were no significant changes in all 22 subjects. However, the LF/HF ratio was also significantly altered in women for the yellow laser stimulation during and after laser irradiation (Figure 5).

**Figure 6:**
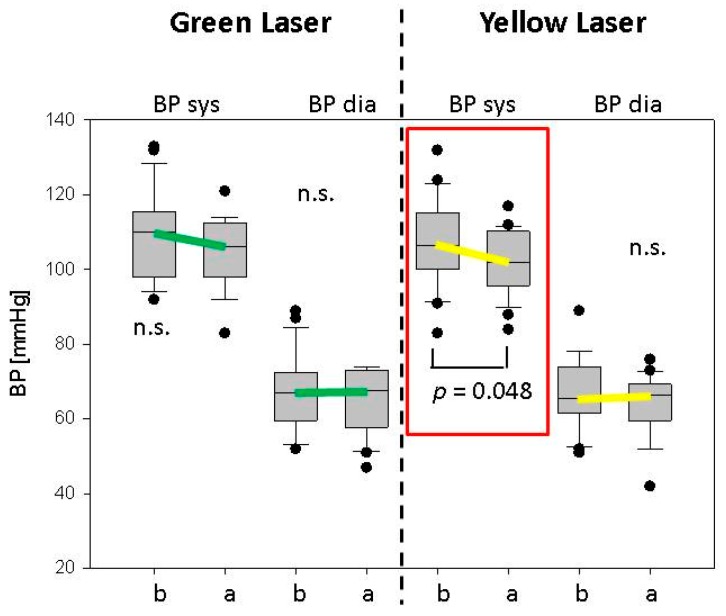
Blood pressure results of the 22 subjects before and after green or yellow laser stimulation. Note the significant decrease in systolic values (BP sys) after yellow laser stimulation at the ear point Shenmen (red rectangle).

The results of the blood pressure measurements were characterized by a significant decrease (*p* = 0.048) of the systolic blood pressure (BP sys) after yellow laser stimulation, whereas the diastolic value (BP dia) showed no significant change. The results of blood pressure readings for both the green and yellow lasers of all 22 subjects are shown in Figure 6.

**Figure 7:**
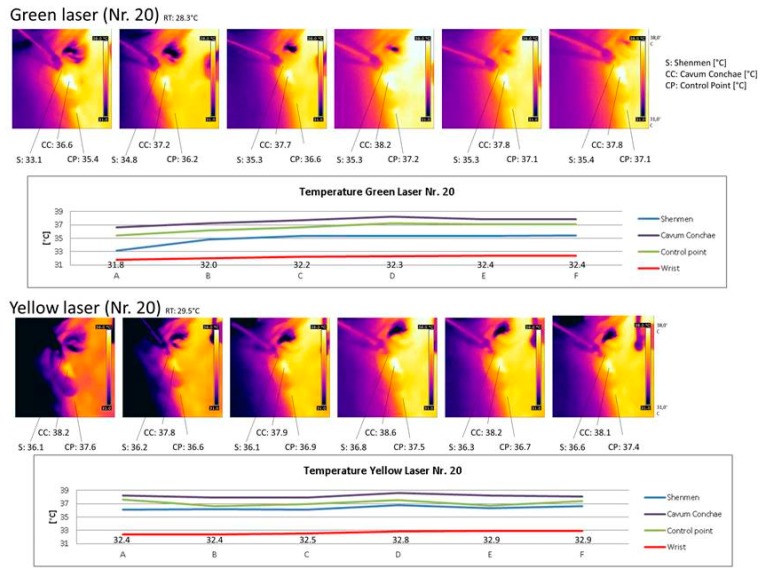
Temperature images and values after green and yellow laser stimulation.

With regard to the temperature measurements, there was no significant change at the measuring points in the area of the ear (Shenmen (S), Cavum Conchae (CC) and Control Point (CP)) after yellow laser stimulation, whereas the green laser showed partially significant temperature increases. An example of the measurements (No. 20) is demonstrated in Figure 7.

The findings of the blood flow velocity (v_m_) in the middle cerebral artery showed no significant changes compared with baseline values (v_m_ before green laser: 62.9 ± 11.6 cm/s; v_m_ after green laser: 63.9 ± 12.9 cm/s; v_m_ before yellow laser: 62.4 ± 12.0 cm/s; v_m_ after yellow laser: 62.2 ± 12.9 cm/s).

## 4. Discussion

As mentioned in the introduction, the original aim of the present randomized crossover study was to analyze the possible effects of green and yellow laser on physiological parameters in human subjects. 

The HR and HRV responses revealed results which are not easy to explain. After laser application, there was a decrease in HR and an increase of the LF/HF ratio. When the LF/HF ratio is increased, it usually indicates that the sympatho-vagal balance has shifted towards sympathetic activation; however, HR did not increase.

The gender-specific evaluation also brought very interesting results. For some time now, the differences between women and men in medicine have no longer been ignored [5]. It is now well known that the individual needs of each patient must be addressed personally, and of course be gender-specific.

Men and women differ physiologically in physique, hormone balance, as well as mental and social factors [6]. In the past, many therapies and medications for men have been designed and developed; however, surprisingly, the gender factor has been largely ignored in acupuncture treatment so far. It is only recently that gender has become an important study factor in acupuncture research [7], and different psychophysiological and neural effects of acupuncture treatment in men compared to women have been shown [8]. Yeo and co-workers [8], for instance, found that women reported a greater intensity of aching compared to men after GB34 acupuncture; moreover, women exhibited greater brain activation in many brain areas, i.e., in the right sided postcentral gyrus, precentral gyrus, precuneus, postcentral gyrus, inferior parietal lobule, declive, middle occipital gyrus, and parahippocampal gyrus, relative to men, after GB34 acupuncture. However, these preliminary gender differences need to be replicated in future research. Therefore, it is not surprising that there are, for example, in the context of laser acupuncture, very few scientific papers that deal with the gender issue [9].

As early as 2004, for example, Litscher and co-workers reported significant gender-specific differences in the perception of pain for both cold and heat [9]. The limits for the painful feeling of cold differed as significantly between men and women in the study mentioned as those for the painful sensation of warmth. Also worth mentioning is the fact that, on average, the tolerance for cold pain stimuli after laser acupuncture (red laser, 685 nm) was higher in women than before the laser treatment [9].

In the training courses on traditional Chinese medicine, there is basically no difference between women and men in terms of treatments [10]. Although an individual diagnosis is carried out, almost no gender-specific therapy is proposed (exceptions: infertility or sexuality). There are hardly any descriptions according to which acupuncture points would have different effects on women or men.

The authors of the present study see for the further development of laser acupuncture the need for an individual approach by medical doctors and therapists. This not only concerns the sex, but of course the age, the constitution, and also the stage of the respective disease. However, an individual adaptation to laser acupuncture can only take place if the scientific basis for it—at least in its basic features—has been clarified, something which has not yet been completely done. Therefore, there is still a lot of experimental work needed to identify the intensity and dose or the frequency of the laser therapy method for the patients on an individual basis.

This work represents a first attempt to quantify the effects of green and yellow laser acupuncture. Nevertheless, there are some limitations in this study. A so-called crossover study design was used. There was no control group (for example, deactivated laser), which is usually included in the field of acupuncture research. Wherever possible (continuous monitoring of the ECG), the temporal aspects were included in the analyses (HR, HRV). Unfortunately, no continuous non-invasive blood pressure measurement or continuous temperature imaging was available for the measurements, so not all parameters were always recorded at the same time (compare Figure 3). Therefore, even an exact comparative time-dependent analysis makes little sense.

The outlier data points in Figure 4 and Figure 5 were included in the analysis. In addition, the experiment was not directly conducted under controlled respiration. The only thing indirectly controllable was that the subjects were asked to breath regularly. However, it has been well documented that HRV parameters (especially frequency domain indices) are profoundly affected by the respiration frequency. It is well known that a frequency power spectrum can be skewed when the respiration frequency falls below 0.15 Hz. As a result, HF and LF powers can be significantly altered. In future investigations, we will also analyze the respiratory frequency, but, as mentioned before, this was not possible in this analysis. 

The autonomic nervous system is a key regulator of the cardiovascular system. The two divisions, the sympathetic and the parasympathetic systems, have co-regulatory effects on cardiac homeostasis. Neurovegetative modulation and dysfunction are also believed to affect various cardiac diseases. Recently, evidence has been provided suggesting gender differences in autonomic nervous system activity [11].

In multiple previous studies, neurovegetative states were primarily assessed using heart rate variability, muscle sympathetic nerve activity, coronary blood flow velocity, and plasma biomarkers. As mentioned before, heart rate variability is a non-invasive measure, which can be analyzed in terms of low-frequency and high-frequency oscillations, which indicate the sympathetic and parasympathetic tone, respectively. These measures have been studied in females and males in states of rest and stress and in cardiac disease [11].

Recent studies support the concept of a significant gender difference in neurovegetative activity. Further studies are necessary to elucidate specific differences and mechanisms, which could guide targeted therapies.

## 5. Conclusions

The following conclusions can be drawn from this pilot study: gender-specific differences in the effects of laser acupuncture likely exist and need to be scientifically proven. The pilot study showed differences in female and male subjects after yellow laser stimulation in the ear. The differences were found primarily in the behavior of the heart rate and its variability. Interestingly, the yellow laser stimulation at the ear point Shenmen was able to lower the systolic blood pressure of the subjects. Although this was also the case with the green laser, significance was found exclusively with the yellow laser. One reason for this could be the higher energy dosage used with the yellow laser.

In order to find explanatory models or mechanisms for the results, comprehensive studies—in animal and human medicine—are certainly necessary.

## References

[1] Bahr F., Litscher G. (2018). Laser Acupuncture and Innovative Laser Medicine.

[2] Round R., Litscher G., Bahr F. (2013). Auricular acupuncture with laser. Evid. Based Complement. Alternat. Med..

[3] Yan J. (2006). Skills with Illustrations of Chinese Acupuncture and Moxibustion.

[4] Task Force of the European Society of Cardiology and the North American Society of Pacing and Electrophysiology (1996). Heart rate variability. Standards of measurement, physiological interpretation, and clinical use. Eur. Heart J..

[5] Miemietz B. (2013). Medizin und Geschlecht: Perspektiven für Lehre, Forschung & Krankenversorgung.

[6] Ngun T.C., Ghahramani N., Sánchez F.J., Bocklandt S., Vilain E. (2011). The genetics of sex differences in brain and behavior. Front. Neuroendocrinol..

[7] Lee S.H., van den Noort M., Bosch P., Lim S. (2016). Sex differences in acupuncture effectiveness in animal models of Parkinson’s disease: A systematic review. BMC Complement Altern. Med..

[8] Yeo S., Rosen B., Bosch P., van den Noort M., Lim S. (2016). Gender differences in the neural response to acupuncture: Clinical implications. Acupunct. Med..

[9] Litscher G., Wang L., Huber E., Schikora D., Schwarz G. (2004). Quantitative Bestimmung geschlechtsspezifischer thermischer Empfindungs- und Schmerzschwellen vor und nach Laserstimulation. Biomed. Tech..

[10] Litscher G. (2012). Are female and male meridians different?. Some spontaneous thoughts. Med. Acupunct..

[11] Pothineni N.V., Shirazi L.F., Mehta J.L. (2016). Gender differences in autonomic control of the cardiovascular system. Curr. Pharm. Des..

